# Supplementary material for: Are carbon emissions trading and green financial instruments synergistic? -Comprehensive quantitative research based on content analysis
Source: PLoS One. 2024 Mar 7;19(3):e0298601. doi: 10.1371/journal.pone.0298601 (PMC10919601; doi:10.1371/journal.pone.0298601)
Supplement: S1 Data — (ZIP) [file pone.0298601.s001.zip › Data/地级市Z_F工作报告.zip!/╔╜╬≈/╜·╓╨╩╨/└┤╫╘╣1⁄2╓┌║┼í╛┬φ┐╦╩2╛▌═°í┐_2011.docx]

各位代表：

　　现在，我代表市人民政府向大会作报告，并就《晋中市国民经济和社会发展第十二个五年规划纲要（草案）》作说明，请予审议，请市政协委员和其他列席人员提出意见。



　　一、“十一五”时期国民经济和社会发展回顾

　　“十一五”时期是我市发展进程中极不平凡的五年。面对国际金融危机和自然灾害频发的影响，面对经济结构深层矛盾的重重困扰，面对保增长保民生保稳定的繁重任务，在省委、省政府和市委的坚强领导下，紧扣“加快科学发展、构建和谐晋中”主题，扎实推进转型发展、安全发展、和谐发展，奋力实施赶超战略，全市经济社会保持又好又快发展，各项工作取得的成就。

　　五年来，我们致力于赶超发展，实现了综合实力的跨越提升。牢记发展第一要务，积极应对复杂变化的形势，认真落实保增长、调结构、惠民生、创环境等一系列政策措施，确保了全市经济社会平稳较快发展。全市地区生产总值年均增长12.1%，2010年达到763.8亿元，是“十五”末的2.3倍；财政总收入年均增长22.8%，达到147.1亿元，是“十五”末的2.8倍；一般预算收入年均增长27.5%，达到64.8亿元，是“十五”末的3.4倍；全社会固定资产投资年均增长26.8%，达到495亿元，是“十五”末的3.3倍；社会消费品零售总额年均增长20.8%，达到277.6亿元，是“十五”末的2.4倍；外贸进出口总额五年累计完成20.4亿美元，比上个五年增长47.8%；2010年全市城镇居民人均可支配收入达到16379元，比2005年增加8346元；农民人均纯收入达到5808.7元，比2005年增加2493.7元；这两项收入年均增幅均高于全省平均水平。经过五年艰苦努力，我市各项主要经济指标实现翻番，在全省的位次前移，跻身于中游行列！

　　五年来，我们致力于调整结构，打开了产业转型的崭新格局。坚持“夯实一产、主攻二产、大兴三产”，努力构建晋中特色的现代产业体系。现代农业扎实推进。实施优势农产品区域布局规划，率先在全省启动现代农业示范区建设，全面提升农业产业化水平。连续五年，粮食生产超过13亿公斤，蔬菜和肉蛋奶总产量稳居全省首位。销售收入超亿元的龙头企业达到13个。农民专业合作社发展到5057个，无公害农产品、绿色食品和有机食品认证累计达到528个，保持全省领先地位。新型工业化进程加快。全面落实八大产业调整振兴规划，工业总产值、销售收入突破千亿元大关。产业集中度大幅提升，煤炭资源整合和企业兼并重组基本完成，全市煤矿单井年产能由不足10万吨提高到87万多吨，总产能由7642万吨提高到1亿吨以上。多元发展格局初步形成，机械装备工业加快升级，煤化工产业延伸发展，电力产业不断扩张，冶金工业结束有铁无钢历史，食品工业加速成长，吉利新能源汽车、中航特种装备及车辆、上海百成纯电动车项目成功引进，迈出了晋中汽车制造的历史性步伐。优势企业发展壮大，五年建成投产项目330项，新增销售收入420亿元，全市销售收入亿元以上企业达到175户，比2005年增加108户，其中两户企业销售收入超过百亿元。工业园区健康成长，安泰集团国家级循环经济试点企业、中煤九鑫省级循环经济园区相继建成，7大重点工业园区实现销售收入283.7亿元，比“十五”末翻了一番半。工业经济效益综合指数达到191.6%，工业新型化水平由30.8%提高到40.08%。服务业布局全面拉开。深入实施“3+7”战略，重点发展现代物流、文化旅游和房地产三大产业，规划和铺开涉及钢材、煤机、图书、粮食、汽配、商贸等领域的21个重大物流项目，全市物流业增加值年均增长13.5%；文化与旅游产业互动发展，旅游接待人数和综合收入年均增长15.76%、35.8%。房地产业发展升温，五年完成投资281.8亿元。



　　五年来，我们致力于统筹城乡发展，推动了城镇化进程不断提速。城市建管同步推进。连续开展城乡基础建设年活动，五年制定城市总体规划、专业规划、控制性详规200余项，完成村镇规划及新农村规划1000余项。全市开工市政重点工程621项，完成投资121.4亿元。2010年底，全市城镇化率达到44.5%,平均每年提高1个百分点。城市功能日益完善。深入开展“四城联创”活动，全市新建污水处理厂9座，建成生活垃圾处理厂10座，新增集中供热面积3092万平方米，污水处理率、垃圾处理率和集中供热普及率五年分别增加58个、22.4个和53个百分点，达到90%、22.4%和77%。市城区和11个县（区、市）创建省级卫生城市并通过验收，市城区、左权县、灵石县被命名为省级园林城市，介休市通过国家园林城市初验。同城发展迈开步伐。坚持主动融入，错位发展，积极推进晋中太原同城化建设，完成了北部新城总体策划、控制性详规等一批重大规划项目，铺开北部新城基础设施工程12项,完成投资16.8亿元，山西高校新校区、物流园区、晋商公园等重大项目建设顺利，城市公交、城市路网、煤气供应、广播电视有线网络同城对接取得积极进展。新农村建设扎实有效。各级财政累计投入新农村建设资金6.6亿元，“创三优”、“四化四改”工程全面实施，500个村达到“十有十个一”标准，建成园林村823个。“五个全覆盖”工程全部超额完成！



　　五年来，我们致力于强基蓄劲，奠定了赶超跨越的坚实基础。强力推进项目建设。五年累计实施627项投资5000万元以上的重点项目，完成项目投资800亿元，带动全社会固定资产投资完成1707.2亿元，是上个五年投资总和的3.7倍。全力扩大招商引资。加强对外开放与交流合作，积极参加重大招商引资活动，共引进项目550个，总投资1748.7亿元，到位资金922.6亿元，累计完成投资813.1亿元，引资到位连续三年全省排名第一。加大城乡基础设施建设力度。路网框架进一步完善，太中银铁路、太长高速晋中段、汾邢高速汾平段建成通车，大西客专晋中段、龙城高速、阳黎高速开工建设。五年累计完成公路交通投资183.8亿元，实施公路建设规模9260公里，新增二级以上高等级公路853公里，全部实现成倍增长，全市“六纵六横”公路网骨架初步形成。兴水改水成效显著，榆社双峰水库续建工程竣工投用，寿阳松塔水电站、灵石石膏山水库、和顺恋思水库建设进展顺利，12座中小型病险水库完成除险加固，解决了134.2万人的安全饮水，新增和改善节水灌溉面积52万亩。电力保障更加有力，全市发电装机容量达到216.6万千瓦，是“十五”末的1.7倍；电网建设五年累计投资42.8亿元，再造了一个晋中电网。加强环境保护和生态建设。狠抓节能减排，搬迁取缔国道省道沿线污染企业87户，万元GDP能耗下降25.62%，超任务0.62个百分点，节能总量420.7万吨标准煤；二氧化硫和化学需氧量减排超额完成任务。2010年市城区空气质量优良率达到98.6%。坚持“生态建设产业化，产业发展生态化”，大搞造林绿化，五年新增有林地面积192.06万亩，林木绿化率提高6.2个百分点，达到30.5%，森林覆盖率20.4%，高于全省2.61个百分点。

　　五年来，我们致力于创新创优，形成了保障有力的体制环境。加强和改进政府工作。推进民主法制建设，自觉接受人大的法律监督和政协的民主监督，积极支持各民主党派、工商联、无党派人士参政议政，五年来市政府及各部门共办理人大代表意见、建议和议案746件，政协委员提案1738件。完善政府工作制度，加强政府法制建设，建立政府应急管理体系，大力开展知责、履责、问责教育，引深反腐倡廉工作，推行基层社会网格化管理，政府执行力、公信力进一步提高。全面推进“法治晋中”、“平安晋中”建设，积极开展联动大接访，加强社会治安综合治理，社会保持和谐稳定。全面推进各项改革。实施国企改革攻坚，晋华、锦纶、轻机三户政策性破产企业改制完成；引深投融资体制改革，公共财政体制建设、国库集中支付、政府采购和工程项目招投标制度、重点工程代建制逐步完善；推进农村各项改革，集体林权制度、农村土地流转改革在全省领先；完成政府机构改革、文化体制改革阶段任务，积极推进事业单位人事制度改革和医药卫生体制改革；推进创新型城市建设，重点开展创新型企业试点、产学研技术联盟试点，科技对经济社会发展的支撑作用日益增强。全面优化发展环境。政务环境建设成效明显，建立了12345便民服务和企业宁静生产日制度，构筑了网上办公、审批、监督平台，完善了市、县、乡、村政务服务网络，在全省率先建立移动电话会议平台和电子政务系统，行政效能和服务效率全面提升。金融生态环境显著改善，组建晋中商业银行，引进股份制银行，建立政策性担保体系，大力发展商业性小额贷款公司和村镇银行，积极探索投融资平台建设，形成了比较完善的区域金融体系。扎实开展一流的城乡基础、法制、安全、生态和人才成长等环境创建活动，环境竞争力进一步提升。

　　五年来，我们致力于和谐发展，促进了民生民利的持续改善。五年财政累计投入民生领域278.7亿元，是上个五年的3.3倍。加快教育均衡发展和学校布局调整，形成了3所市属大专院校、30所普通高中、20所中等职业学校、1000所义务教育学校的发展格局。完善城乡卫生医疗体系，新建55个城市社区卫生服务中心和服务站，县乡村三级医疗机构达标率97.05%，新农合参合率95.94%，基金使用率94.48%。扩大城乡就业和社会保障，建立了“九个一批”就业模式，五年新增城镇就业20.49万人，各类保险参保人数、保费征缴额逐年提高，职工失业和工伤保险实现市级统筹，城乡低保实现应保尽保。深入开展精神文明创建活动，加强基层文化建设，农村广播电视覆盖率100％，率先在全省实现新农村健身场地基本覆盖，人均公共体育场地面积由“十五”末的0.98平方米增加到1.6平方米。加大扶贫开发力度，五年减少贫困人口16.49万人。坚持不懈狠抓安全生产，安全生产形势稳定好转。



　　与此同时，我们大力支援南方抗击雨雪冰冻灾害，扎实开展汶川特大地震救援工作，全力防范甲型H1N1流感疫情。特别是2009年，面对国际金融危机带来的困难和挑战，深入落实保增长、保民生、保稳定措施，在全省第一家出台帮助工业企业克服困难加快发展的31条减、免、缓、停措施，有效促进了全市经济尽早企稳回升，以出色的工作经受住了重大事件和复杂形势的考验。

　　五年来，我们先后荣获全国双拥模范城、义务教育均衡发展先进地区、农村公路建设示范工程先进单位、社会治安综合治理“长安杯”，省和谐社会建设先进市、节能环保优秀奖、现代农业建设先进市、新农村建设先进市、安全生产先进单位、省级卫生城市等荣誉称号，多项工作实现创新突破，走在了全国、全省的前列。

　　各位代表，我向大会郑重报告，我市“十一五”规划目标任务全面完成，向全市人民承诺的实事全部兑现，晋中进入了一个厚积薄发、跨越崛起的发展新阶段！

　　五年的实践充分表明，办好晋中的事情，必须始终从晋中的发展基础和阶段特征出发，坚持以加快发展为第一要务，狠抓项目建设，以卓有成效的项目建设强基础、促转型、增后劲；必须始终坚持统筹兼顾的根本方法，促进城乡协调，突出改善民生，让发展成果最大程度地惠及广大群众；必须始终高度重视安全生产和社会稳定，把转型发展、安全发展、和谐发展与赶超发展统一起来，全力营造安全、稳定、和谐的发展环境；必须始终保持强烈的机遇意识、创新意识和争先意识，在全国、全省的大坐标中寻找差距、定位目标，推动工作的创新突破和高效落实；必须始终把推进改革开放和创优发展环境贯穿到发展全过程，引进外力、激活内力、凝聚合力，形成共谋发展、共创事业的生动局面。这些成功经验和精神财富，必将激励我们在新的发展征程上不断前行！



　　五年的成绩来之不易，这是省委、省政府和市委坚强领导的结果；是市人大、市政协监督支持的结果；是社会各界共同努力的结果；是全市人民万众一心、团结奋斗的结果。我代表市政府，向全市人民、各民主党派、各人民团体和社会各界人士，向驻地部队、武警官兵和国家、省驻地单位，向所有关心支持晋中发展的海内外朋友们，表示崇高的敬意和衷心的感谢！



　　我们也清醒地认识到，我市经济社会发展还存在着不少困难和问题：发展不足、投资不足、后劲不足的问题依然突出；结构调整、产业升级、节能减排任务艰巨；体制机制创新不够，发展环境还不宽松；社会事业和民生建设相对薄弱，社会管理和服务水平亟待提高，城乡居民收入不高、增长不快；政府职能转变相对滞后，部分干部的思想观念和工作作风与新形势、新任务的要求还不相适应。



　　各位代表，成绩属于过去，未来充满艰辛。站在“十二五”新的发展起点上，我们既感到巨大压力，也感到百倍信心。我们有决心、有能力战胜困难、破解难题，推动全市经济社会在又好又快的道路上加速前进，决不辜负全市人民的期望和重托！



　　二、“十二五”时期主要目标和任务

　　“十二五”时期，是全面建设小康社会的关键时期，是深化改革开放、加快转变经济发展方式的攻坚时期，也是实现转型跨越发展的重要时期，我市经济社会发展面临着难得的机遇。从全国看，随着国家扩大内需和促进中部崛起战略深入实施，能源、材料和装备的需求将持续增加，对资源型地区经济发展产生积极的拉动作用。从全省看，国家资源型经济转型综合配套改革试验区加快建设，必将为我市先行先试、转型跨越提供有力的政策支持；全省“一核一圈三群”城镇群建设逐步加快，有利于我市接受太原辐射、承接产业转移、完善功能配置，加快区域一体化发展。从全市看，我市资源、区位和成本优势独特，投资环境良好，为我市大规模引进项目、资金、先进技术和优秀人才创造了有利条件；人均GDP突破3600美元，进入工业化、城镇化加速发展的新阶段，发展的内生动力逐步释放。特别是全市干部群众思想统一，人心思上、干事创业的热情高昂，只要我们牢牢把握机遇，振奋精神，勇于争先，埋头苦干，负重拼搏，一定能够创造新的成就，谱写新的篇章！



　　根据市委二届六次全会精神及“十二五”规划《建议》，市政府编制了“十二五”规划《纲要（草案）》，明确提出“十二五”时期，要坚持以科学发展为主题，以加快转变经济发展方式为主线，以建设全省“四化”率先发展区为统领，按照转型发展先行区、晋商文化复兴地、现代宜居生态市的战略定位，实施经济转型、赶超发展两大战略，做大经济总量，提高发展质量，做强八大产业，着力改善民生，加快建设新型化工业强市、现代化农业大市、文化旅游名市、城镇化新市，努力建成全省最重要的装备制造中心和新型煤化工基地、中部地区现代物流枢纽、全国优秀旅游目的地，力争提前一年实现主要指标翻番。

“十二五”时期经济社会发展的主要目标是：

　　——主要经济指标翻番。全市六项主要经济指标实现翻番，力争提前一年实现，总量和人均占有量跻身全省上游。其中，地区生产总值年均增长15%，力争17%；财政总收入、一般预算收入年均增长18%，固定资产投资年均增长30%以上，城镇居民人均可支配收入年均增长15%，农民人均纯收入年均增长15%以上。

　　——转型发展先行突破。做大做强物流金融、装备制造、新材料、煤化工、现代农业、文化旅游、电力和煤焦等八大产业，煤焦产业比重大幅下降，产业结构明显优化。到“十二五”末，建成山西规模最大、功能最全的物流园区，建成技术领先、机制灵活的现代农业示范园区，建成10个循环发展、效益明显、销售收入过100亿元的工业园区，将文化旅游业、现代物流业打造为全省的“龙头”，力争装备制造业、煤化工、现代农业站在全省前列。

——特色优势全面彰显。加快晋中太原同城化进程，推进城镇集群化发展，“一小时经济圈”基本形成，“108综合发展廊带”综改集中试验区建设取得全面突破，建设太原都市区最具活力和发展潜力的板块，打造太原都市圈核心增长极，实现中心城市、城镇群、经济圈互促互动，生态休闲、宜居宜商城市特色进一步凸显，市域发展比较优势得到全面提升。

　　——努力建设幸福晋中。居民收入增长与经济发展同步，劳动报酬增长与劳动生产率提高同步，社会保障水平明显提高，覆盖城乡的基本公共服务体系更加完善，民主法治更加健全，精神文化生活更加丰富，社会更加和谐稳定，人民群众生活的安全感、舒适感、幸福感全面提升。

　　“十二五”时期经济社会发展主要任务是：

　　——加快建设新型化工业强市。实施工业强市战略，坚持大产业支撑、大企业引领、大项目带动，突出煤炭产业升级和装备制造业扩规，扎实抓好“百亿产业、百亿企业、百亿园区、百亿集群”工程，不断壮大中小企业实力，力争“十二五”时期全市规模以上工业增加值年均增长19%以上，形成结构优化、技术进步、清洁安全、附加值高、吸纳就业能力强的产业体系；加快中国纺机名城、液压元器件国家示范基地和全国玻璃器皿生产出口基地建设，打造全省重要的装备制造中心和新型煤化工基地。



　　——加快建设现代化农业大市。按照“一村一品、一县一业”思路，大力推进传统农业向现代农业、传统农村向现代城镇、传统农民向新型农民“三个转型”，倾力实施粮食优质化、蔬菜设施化、养殖工厂化、林果集约化、加工品牌化、生态产业化“六大工程”，建设全省最大、全国精品的绿色蔬菜生产基地，华北一流、全国知名的畜产品生产加工基地和省城居民观光休闲基地。力争全市农业总产值年均增长15%，农业科技进步贡献率每年提高2个百分点。

　　——加快建设文化旅游名市。深入挖掘文化内涵，推进文化体制改革，打造特色文化品牌，大力发展文化事业和文化产业。到2015年，力争全市文化产业占服务业增加值的比重达到6%以上，走在全省前列。坚持文化与旅游有机融合、互动发展，实施“大集团、大景点、大服务”战略，打造核心板块和精品线路。到2015年，全市旅游综合收入达到300亿元，力争文化旅游产业综合发展水平进入中部十强市，建成全国优秀旅游目的地。

　　——加快建设城镇化新市。以晋中太原同城化为龙头，按照“一轴两心两区四联”城镇空间布局结构，做大市城区，打造“大县城”，发展小城镇，建设“中心村”，整体推进城镇群建设；提高城乡建管水平，构建城乡统筹、布局合理、区域协调、环境友好、功能完善的城镇化发展机制。到2015年，建成国家级园林城市、省级宜居城市，力争完成国家级卫生城市、省级文明城市创建，全市城镇化率达到55%。

　　——加快建设现代宜居城市。围绕建设资源节约型、环境友好型社会，牢固树立“绿色、低碳、洁净、健康”发展理念，大搞造林绿化，发展循环经济，狠抓治污减排，强推节能降耗，率先将我市建成经济生态高效、环境生态优美、社会生态文明的幸福家园。到2015年，全市森林覆盖率达到25%以上，万元GDP综合能耗比“十一五”末下降16%左右，市城区空气质量稳定保持国家二级标准。

　　各位代表，建设全省“四化”率先发展区，是时代发展的要求，是人民赋予的重任。我们坚信，只要全市干部群众万众一心、扎实苦干，争为三晋先、敢为三晋先，我们的目标就一定能够实现，一个崭新的晋中一定会展现在世人面前！ 

　　三、2011年主要工作

　　2011年是“十二五”规划的开局之年，也是建设全省“四化”率先发展区的起步之年，做好全年各项工作意义重大。我们要深入贯彻落实市委、市政府《关于加快建设全省“四化”率先发展区的决定》，积极在国家资源型经济转型综合配套改革试验区建设上先行先试，抓实结构调整、项目建设、现代农业、城乡统筹、生态绿化、改革创新、民生改善、社会管理等各项工作，推动转型跨越发展迈出坚实步伐。

　　今年主要预期目标是：地区生产总值增长13%；规模以上工业增加值增长20%；全社会固定资产投资增长25%；财政总收入增长18%以上；外贸进出口总额增长25%；社会消费品零售总额增长18%；城镇居民人均可支配收入增长13%；农民人均纯收入增长12%以上。确保完成以下约束性指标：万元生产总值综合能耗下降3.5%左右；完成省下达六项污染物减排任务；居民消费价格涨幅控制在4%左右；城镇新增就业2.97万人以上，城镇登记失业率控制在4.2%以内。



　　重点抓好以下八方面工作：

　　（一）深化经济结构调整，全力加快产业转型步伐

　　坚持以煤为基、多元发展，加快构建具有晋中特色的现代产业体系，促进经济转型发展。



　　改造提升传统产业。坚持循环发展，延长产业链条，对煤、焦、冶、电四大产业进行改造提升。做好煤矿企业兼并重组整合后续完善工作，加强矿井基建技改，进一步夯实矿井安全质量标准化建设，严格规定程序推动复工复产等重点工作，年内力争80座以上基建技改矿井开工建设。做好焦化行业兼并重组，上新上大，淘汰落后，已通过预核准的3个焦化项目力争年内开工建设。积极发展H型钢、高碳铬铁、镍铬合金等冶金新产品，增强产业竞争力。大力发展大型空冷燃煤发电机组和热电联供，争取中煤寿阳煤气化循环发电项目落地，华能左权煤电一期工程具备投产条件。



　　培育壮大新型优势产业。把发展非煤、非资源型产业作为结构调整的重点，围绕建设全省最重要的装备制造中心和新型煤化工基地，促进一批新项目落地和做大做强。大力发展以新能源汽车为龙头的装备制造，力争吉利新能源汽车、中航特种装备及车辆、太重液压设备重大转型项目上半年开工。积极延伸煤化工链条，加快昔阳电石、和顺合成氨·尿素等在建工程进度，推进东方希望4A沸石等项目落地。扩大新材料产业规模，抓好富士康镁及镁合一体化项目，加快恒天碳纤维原丝产业化发展和平遥煤化新型光学材料、三佳有机硅二期、聚源硅微粉等项目建设。抓好食品工业与农业产业化的协调发展，重点推进祁县燕京啤酒、寿阳雨润肉制品加工、今麦郎饮品生产等项目建设。

　　加快发展现代服务业。突出抓好现代物流和文化旅游产业。立足打造全省现代物流核心区和中西部物流中心，加快山西能源工业国际分销物流港、山西粮食物流中心等项目建设，全力推进太铁货运物流中心、富士康科技园、方略保税物流港等前期工作。瞄准文化旅游名市目标，抢抓平遥古城列入全省三大重点开发景区的机遇，认真落实市三届人大常委会第五次会议审议意见，按照晋商文化、民俗文化、红色旅游、休闲度假总体布局，加强基础设施建设，完善要素体系，提高管理水平，提升服务质量；强化文化旅游名市宣传，在国内外主流媒体推出我市主体文化形象，打造经典文化品牌和精品旅游线路；加快平遥古城旅游服务基地提档升级，抓好石膏山风景区、静升古镇开发、榆次老城三期、晋中旅游直升机场、华夏文化遗产主题公园等重大项目建设，力争全市旅游总收入达到130亿元，旅游业增加值占GDP比重再增加1个以上百分点。推进房地产业健康发展。加快发展商贸、金融、信息等服务业。



　　推进园区建设和产业集群发展。大力推动产业、企业、项目和要素向园区集聚，提升产业集中度和园区承载力、带动力。规范提升现有7大工业园区，抓好山西新能源汽车·机械装备制造区、液压工业园、东方希望铝工业循环园等产业规划、项目引进和整体推进工作。整合区域特色产业，推进纺机、玛钢、玻璃器皿、老陈醋、农产品加工等产业集群发展。

　　各位代表，转型发展是“十二五”的主题和最鲜明特征。我们要重点在产业转型上着力，加快实现传统产业新型化、优势产业规模化、新兴产业高端化、支柱产业多元化，努力走出一条资源型地区转型发展的新路子！

　　（二）加大项目建设力度，增强经济发展后劲

　　坚持重点项目和重点工程储备、签约、落地、实施“四位一体”，统筹推进。

　　做实项目盘子。加强政策研究和市场分析，做好项目生成、筛选、策划工作，提高项目储备数量、质量和成熟度。优化项目结构，入库项目中非煤产业项目占到60%以上。实施动态管理，形成谋划一批、储备一批、发布实施一批的良性循环。

　　大力招商引资。积极参加“中博会”、“渝洽会”、“厦洽会”等重大招商活动，围绕煤化工、机械装备、新材料、金融物流、现代农业、文化旅游等领域，进行专题招商和点对点招商。充分发挥企业招商引资主体作用，实行以商招商、定向招商，提高招商引资针对性和实效性。全年新引进项目总投资1000亿元以上。

　　狠抓项目落地。落实最严格的耕地保护制度和节约用地制度，积极用好批而未供土地，引导使用存量土地，扎实推进增减挂钩试点，主动盘活矿山企业闲置土地。推行排污权有偿使用和交易，用好用足环境容量。认真落实鼓励引导民间投资的政策措施，特别要引导煤炭资源整合退出资本积极投入非煤领域，构筑项目争资、招商引资、民间投资、银行融资等多元化的投融资机制。各级各部门实行并联工作、高效服务,一对一解决项目前期的突出问题，确保一批成熟项目尽快落地。

　　加快建设进度。强化市县两级对重点工程建设的综合协调调度，实行每月会商、联审、督查、通报的工作机制，加快推进各项重点工程特别是37项省级重点工程建设。完善市县领导包项目责任制，督促项目牵头单位和建设主体单位抓开工、抓进度，做到“以旬保月、以月保季、以季保年”。全年实施重点工程570项，年度计划投资500亿元。

　　各位代表，项目和投资是落实“十二五”规划的关键，今天的项目就是明天的发展，今天的投资就是明天的产出。我们一定要把项目建设作为保增长、调结构、促转型的第一要务，确保完成年度投资目标！

　　（三）大力发展现代农业，多渠道增加农民收入

　　扭住农民增收目标，突出“一村一品、一县一业”，用工业化理念组织农业，加快现代化农业大市建设步伐。



　　加快现代农业示范区建设。整体推进园内五大基地、四项基础工程和四大支撑体系建设，重点抓好30个示范项目开工建设，年内完成投资10亿元以上。

　　大力发展“一村一品”、“一县一业”。强化规划引导和政策扶持，统筹推进6个基地县、30个示范乡镇建设，年内完成20个高标准粮食专业村、100个蔬菜专业村、20个养殖专业村、40个林果专业村、40个原料基地村、20个生态庄园观光村建设，新发展设施蔬菜6万亩、干鲜果23万亩，新建和改扩建规模养殖场200个，推进发展城郊农业、都市农业。



　　全面提高农业组织化程度。以实施“513”工程为抓手，扶持发展农产品加工销售龙头企业，规范提升农民专业合作社，积极培育营销大户和农民经纪人，完善“公司+基地+中介组织+农户+标准化”经营组织体系，提升农业产业化水平，力争农产品加工流通龙头企业销售收入80亿元，打造100个市级示范合作社和50个联合体，辐射带动农户20万户。加快土地流转，推进农产品物流与市场体系建设，搞好产销衔接，提高晋中农产品在太原及周边城市的市场份额和比重。



　　有序推进新农村建设。继续重点抓好228个新农村建设，推进20个新农村片区建设工程。实施农民大培训“六个一”工程，培训农民100万人次，转移农村劳动力5万人。完成1500座改厕任务。继续推进扶贫开发，实施片区开发、整村推进、移民扶贫、产业扶贫、劳动力转移培训、科技教育扶贫六大工程，完成移民搬迁5000人。深入实施“万村千乡市场工程”、“新网工程”和“双三十”农产品市场工程。扎实做好“农民增收明白卡”试点和推广工作。

　　（四）统筹推进城乡建设，加快市域城镇化进程

　　抓住晋中太原同城化机遇，突出规划引领，加快构建“一轴两心两区四联”城镇空间布局结构。

　　举起“同城”龙头。围绕完善城市功能，抓好市城区“百亿市政重点工程”，实施“北进、西联、南扩、东延”扩城计划，全面推进以北部新城、山西高校新校区为重点的北进工程，以榆次工业园二期、现代物流园区为重点的西联工程，以修文工业基地、潇河公园一期为重点的南扩工程，以装备机械制造园区为重点的东延工程，加快与太原互动、互通、互享，共同打造太原都市圈高标准核心区。



　　加强城乡建设管理。加快晋中市城市总规修编工作，同步推进产业规划、土地规划和城市总体规划，加快城乡建设规划全覆盖，启动城市数字化管理。加快大县城开发建设，以3个省级示范镇和15个市级推进镇为重点，搞好特色镇规划建设，因地制宜做大做强“中心村”。深入开展“四城联创”、城乡整洁行动和农村环境“创三优”活动，力争80%乡镇、30%行政村达到市级卫生乡村标准。创新城镇管理体制，做好介休义安镇行政管理体制改革试点工作。加大户籍管理改革力度，引导在城镇稳定就业和居住的农民有序转为城镇居民。全市城镇化率提高2个百分点。

　　强化基础设施建设。铺开水利建设“双百工程”，加快东山供水、松塔水电站等大型骨干工程进度，积极争取引黄南扩工程。抓好龙城、汾邢、阳黎三条高速公路建设，高标准改造县乡公路300公里，开工建设晋中汽车客运总站。落实城乡同网同价政策，启动新一轮农村电网改造工程，进一步改善电网网架结构，建成各级电网协调发展的智能电网。推进“气化晋中”建设，重点抓好煤层气开发，加快东山太原煤层气管网建设。

　　促进县域经济发展。切实加大对县域经济发展的支持力度，适应省直管县财政体制改革要求，积极帮助县（区、市）争取上级专项资金和转移支付；落实“综改试验”扩权强县政策，争取行政审批事项部分下放。充分调动县（区、市）发展积极性，继续完善分组竞赛与单独考核相结合的办法，促进县域竞相发展。大力发展民营经济，实施中小企业成长工程，推进创业孵化基地建设，加快发展劳动密集型产业。

　　各位代表，县域经济是晋中发展的强大支撑，县域兴、晋中兴，县域强、晋中强。各县（区、市）和晋中开发区都要充分发挥优势，加快发展，竞相赶超，为实现全市转型跨越争做贡献，共同建设好我们的美丽家园！



　　（五）加大造林绿化和节能减排力度，积极推进城乡生态化

　　突出增绿、减排、节能、循环，提升生态文明建设水平。

　　大搞造林绿化。围绕创建省级林业生态市目标，弘扬晋中“大造林”精神，加快打造东山、平川双百公里林果带和千里林业生态圈，突出抓好市城区北山生态绿化等11项重点工程，全市林木绿化率增加1个以上百分点。狠抓城乡绿化，争创国家级园林城市1个，完成村庄绿化100个。加大森林资源保护力度，巩固造林绿化成果。

　　狠抓节能减排。继续实施“双百户企业”节能行动计划，推广节能合同管理，加大技改节能力度，开展行业能效对标，加快淘汰落后产能。围绕创建省级环保模范城市目标，严格控制二氧化硫、化学需氧量、氮氧化物、氨氮以及烟尘、粉尘等主要污染物排放总量，严格环境准入制度，确保完成各项减排指标。加快城市垃圾处理厂、污水收集管网和中水回用工程建设，打好以燃煤锅炉与城镇生活污水治理为重点的治污攻坚战。

　　大力发展循环经济。引导和支持重点企业，建设资源循环利用项目，加大安泰、中煤九鑫国家级、省级循环经济试点推进力度。鼓励企业建立循环经济联合体，推广资源循环利用技术，实现能源梯级利用和物料循环使用。推广低碳生产、清洁生产，推动甲醇燃料发动机研发，建设城市电动汽车充换电设施，推广应用节能环保新材料。

　　（六）加快改革创新步伐，在转型综改试验区建设中先行先试

　　抢抓“转型综改试验区”建设机遇，积极鼓励创新，不断深化改革，构建体制机制和科技人才支撑。

　　加快打造创新平台。以“108综合发展廊带”建设为载体，落实好已有政策，创新潜在政策，争取可能政策，学习、移植和推广其它试验区好经验、好做法，在建立资源开发的生态补偿机制、资源型产品价格形成机制、矿区企业分离办社会的投入机制上先行先试，探索项目审批制度、投融资体制等改革。抓好试点县转型综改试验工作，争取全市转型综改行动方案早日获得批准。

　　强化科技人才支撑。增加市县两级科技研发资金占本级财政预算支出比例，实施项目引导、载体建设、资金保障和服务支撑“四大科技工程”，新建11个工业企业研发中心、10个农业研发中心、8个科技成果转化基地、10个产学研合作示范基地，启动建设晋中市公共科技资源共享平台。积极申报纺机、玻璃器皿国家级产品质检中心。加快构建和完善工农业标准化体系。大力引进和培养高端专业技术人才、高层经营管理人才、高级金融人才，加强技能人才培训和农村实用人才培训。

　　切实优化金融服务。继续引进银行、证券、保险等金融机构和金融中介机构，积极推动新型农村金融机构的设立，鼓励地方性金融机构在县级设立分支机构，加快县级农村信用联社改制农村商业银行进程，强化小额贷款公司与金融机构的融资合作，培养优质中小企业在天津股权交易所挂牌交易，抓好筹融资平台和担保体系建设，扩大农村小额保险与“三农”保险的覆盖面。



　　统筹推进各项改革。做好晋华、锦纶、轻机等破产改革后续工作，完成药材公司、格芙兰破产改制，启动金恒化工、晋中建设集团改制工作；搞好乡镇机构改革，基本完成事业单位清理规范和分类工作，完善市县财政管理体制；全面完成医药卫生体制改革五项任务，按时完成基层医疗机构综合改革工作，稳步推进公立医院改革和县域医药卫生一体化综合改革试点；引深农村土地经营制度、城乡水务一体化改革，完成集体林权制度主体改革。

　　各位代表，建设“转型综改试验区”，是我们面临的最重要、最给力的政策支撑和重大机遇。我们要抓住这一难得机遇，敢于先行先试、率先突破，靠改革创新为转型跨越增添强大动力！

　　（七）持续改善民生民利，提升人民群众幸福指数

　　继续加大公共财政对民生的投入力度，抓好以保障改善民生为重点的社会建设，不断完善城乡公共服务体系，集中力量办好关系群众切身利益的好事、实事。今年全市将安排一般预算支出的64.66%用于民生建设。

　　大力发展社会事业。提升教育发展水平，深入贯彻落实国家、省《中长期教育改革和发展规划纲要》，全面启动国家级统筹推进义务教育均衡发展改革试点，扎实抓好“校安工程”扫尾工作，下大力气解决幼儿“入园难”问题，推进义务教育校校达标、职业教育基础能力建设、教育信息化建设和高素质教师队伍建设工程，普通高中与中等职业学校招生比例稳定在1:1，高中阶段教育普及率达到95%以上。推进职业教育集团体制机制创新，支持职业院校与行业企业紧密合作，加快培养应用型技能人才。加强医疗卫生服务和公共卫生服务体系建设，全面实施国家基本药物制度，推进基本公共卫生服务均等化，新农合参合率稳定在96%以上。推动文化大繁荣、大发展，加强文化精品创作，重视文物和非物质文化遗产保护，力争启动市博物馆、图书馆建设。抓好新闻出版、广播影视事业，加快发展文化产业。深入开展“校园文化”、“广场文化”、“企业文化”、“社区文化”活动，推进全民健身工作，办好第三届市运会暨全民健身节，大力实施文化惠民工程，各县（区、市）图书馆、文化馆力争全部建成达标。

　　着力解决民生突出问题。积极扩大城乡就业，开展创业型城市创建活动，市级充分就业社区力争达到10个。继续推行“九个一批”就业模式，改善创业环境，强化就业服务，做好高校毕业生、城镇就业困难人员和农村富余劳动力就业工作，全年创业带动就业9200人，失业人员再就业1.08万人，就业困难人员就业2459人。完善社会保障体系，进一步扩大社会保险和城乡低保覆盖面，加强社保基金征缴和监管，全面启动“社会保障一卡通”工作。建立健全被征地农民社保预存款制度，稳步推进事业单位养老保险制度改革，完成医疗保险市级统筹。进一步完善社会救助体系，落实好农村“五保”等政策，提高孤儿养育资助标准，抓好农村敬老院建设。加大保障性住房建设力度，强化组织领导和部门协调，多渠道筹集建设资金，全年投资44.02亿元，开工建设保障性住房2.87万套、201.64万平方米，含续建项目在内，确保年底前竣工保障性住房1.72万套。加强价格调控和市场监管，建立物价上涨与最低生活保障标准挂钩联动机制，严厉查处恶意炒作、串通涨价、哄抬价格等行为，改善消费环境，大力促进消费。

　　集中办好关系群众切身利益的具体实事。启动新的“五个全覆盖”工程，今年新建农村体育健身场地200个，农村文化室319个，行政村农民健身工程提前实现全覆盖；农村街巷硬化覆盖率达到60%以上；建设农村便民连锁店600个；农村职业教育免费覆盖学生2.8万人；新型农村养老保险覆盖率超过80%；明年全面完成五个全覆盖任务。农村中小学校学生平均公用经费补助标准提高100元。人均基本公共卫生服务经费由15元提高到25元。企业退休人员基本养老金每月人均增加140元。新农合和城镇居民基本医保财政补助标准由人均120元增加到200元。城乡居民最低生活标准人均每月分别增加25元和22元。对2203名贫困残疾人实施康复救助。启动20所城市公办幼儿园和40所乡镇公办中心幼儿园建设。改造城市小街巷30条。

　　各位代表，为人民谋福祉是政府的天职。我们将以更主动的作为和更多的投入，不断提高社会保障水平、收入水平、健康水平、教育水平，让人民群众生活得更有质量、更有尊严、更有幸福感！



　　（八）统筹推进社会建设，维护社会和谐稳定

　　坚持经济社会协调发展，积极探索加强和创新社会管理的新路子，推进和谐晋中建设。

　　加强社会主义民主法治和精神文明建设。认真执行人大及其常委会的决议决定，广泛听取各民主党派、工商联、无党派人士的意见和建议，积极做好人大代表建议和政协委员提案办理工作，自觉接受人大、政协和社会各界的监督。支持人民法院、检察院依法履行职责。加强基层民主建设，保障人民群众民主权利。做好第九届村委会换届选举工作。加快依法治市进程，启动“六五”普法工作，提高全民法制意识。深入推进群众性精神文明创建活动，开展公民教育和未成年人思想道德教育，大力弘扬晋商精神、太行精神、大寨精神和时代创新精神，培育城市文化特色，推进移风易俗，倡导文明新风，引深文明城市创建工作。

　　加强和创新社会管理。以安全监管网格化、社会管理网格化、居民自治网格化为重点，探索建立公共管理体系。强化基层政权组织的社会管理服务职能，以村委会、社区和基层综治信访服务中心为依托，整合综治、司法、行政和信访资源，建立大综治、大调解平台，筑牢加强管理、维护稳定的第一道防线。坚持用群众工作统揽信访工作，强化信访工作责任，对群众反映突出问题实施专项治理，妥善处理征地拆迁、企业改制等引发的社会矛盾。健全突发公共事件应急管理机制，提高应急处置能力。加大食品药品安全监管力度，保障公众饮食用药安全。加强社会治安综合治理，完善防控体系，严厉打击各类违法犯罪。统筹做好人口和计划生育工作。深化全民国防教育和双拥共建活动，推进国防后备力量建设。加强国家安全工作。大力发展社会福利和慈善事业。推进防震减灾。开展爱国卫生运动。抓好外事侨务、人防、民族宗教、方志、档案、保密、对台、气象、科协、残联等工作。发展老龄事业，关爱老年人。重视和支持工会、共青团、妇女儿童工作。 

　　不断提升安全生产水平。全力加强风险管理，全面引入风险管理标准体系，建立全员、全方位、全过程的动态风险管控机制，保障安全生产风险可控、能控和在控。不断创新工作方法，制定具体奖励扶持措施，广泛开展安全创建竞赛活动，通过树典型、立标杆，提高全员安全意识和业务素质，引导激励组织创建本质安全型企业。实施事故责任倒逼机制，落实安全生产“两个主体”责任，严格执行“网格化”监管、逐月隐患排查和安全例会等一系列制度，持续开展安全生产管理年活动，抓好重点领域和重点行业的专项整治，实现责任全覆盖，确保管理制度化、常态化。



　　各位代表，抓好全市安全生产，态度是关键。态度乘以能力等于效果。只要我们拿出百分之百的态度，真正做到尽心、尽责、尽力，就一定能实现安全形势的明显好转、稳定好转和根本好转！

　　四、加强政府自身建设



　　适应建设全省“四化”率先发展区的要求，以更加务实的举措，加快政府职能转变和管理创新，提高执行力，增强凝聚力，赢得公信力，努力建设让人民群众更加满意的政府。

　　推进依法行政。坚持干部学法制度，不断提高行政机关工作人员的法律素养和依法行政能力，严格按照法定权限和程序行使权利、履行职责。完善重大决策公众参与、专家论证、风险评估、合法性审查和集体讨论机制，提升决策科学化、民主化水平。严格落实行政执法责任制，做好规范性文件制定、审查、备案、清理工作，加大行政执法案件查办力度，高质量办好行政复议案件。深入推进政务公开，依法保障人民群众的知情权、参与权、表达权和监督权。



　　提高行政效能。严格执行《市政府工作规则》，优化工作流程，简化办事程序，建立部门协调议事和并联工作机制，推进政府机关高效运转。学习借鉴循环质量管理理念，建立健全“决策—落实—督查—改进—提升”全过程管理体系，推动管理水平持续提升。加强学习培训，全面提高公务员队伍履职尽责的本领和执行能力。完善工作目标责任制，深入开展知责、履责、问责教育活动，形成确保责任落实的有效机制。狠抓作风建设，严明工作纪律，加强效能监察，严厉查处不作为、慢作为、乱作为，切实提高各级行政机关的工作效率。

　　创优政务服务。进一步转变政府职能，提高政府社会管理和公共服务水平，加快建设服务型政府。深化行政审批制度改革，进一步规范和减少行政审批。全面推行服务承诺、并联审批、“一站式”办结和重大项目全程代理服务等制度，积极推行“两集中、两到位”改革，完善网上审批制度，努力把审批减到位、环节压到位、收费降到位。切实加强市、县、乡、村四级政务服务网络建设，加快形成全方位、无缝隙、全天候的行政审批和电子监察服务格局。痛下决心解决影响发展环境的突出问题，加快把全市软环境建设提高到一个新水平。

　　加强廉政建设。严格执行廉洁从政各项规定，认真履行“一岗双责”党风廉政建设责任制，扎实推进廉政风险防控机制建设，严格遵守党员领导干部不大操大办、不参与赌博的承诺。狠抓增收节支，降低行政成本，做到公务用车、会议、公务接待、党政机关人员出国四项经费支出“零增长”。强化预算约束，逐步推进预算公开特别是“三公”经费公开。继续深入开展煤焦领域反腐败专项斗争，强化工程建设、土地使用权出让、矿产资源开发、招投标、政府采购等重点领域监管。加强行政监察和审计监督，严肃查处各类违法违纪行为，进一步健全惩治和预防腐败工作体系，营造风清气正的良好发展氛围。

　　各位代表，建设全省“四化”率先发展区是我们的奋斗目标，再造一个新晋中是全市人民的共同愿望。让我们在省委、省政府和市委的坚强领导下，抓住机遇，迎难而上，团结奋斗，顽强拼搏，全力推动我市经济社会转型跨越发展，努力实现“十二五”良好开局，以优异的成绩迎接中国共产党建党90周年！
